# TIGRIS and EUPHRATES eventually join and provide new evidence: a narrative review of the polymyxin B hemoperfusion

**DOI:** 10.1186/s40560-025-00835-6

**Published:** 2025-11-25

**Authors:** Toshiaki Iba, Julie Helms, Isao Nagaoka, Michio Mineshima, Ricard Ferrer

**Affiliations:** 1 Faculty of Medical Science, Juntendo University, 6-8-1 Hinode, Urayasu, Chiba 279-0013 Japan; 2 Strasbourg University (UNISTRA); Strasbourg University Hospital, Medical Intensive Care Unit - NHC; INSERM (French National Institute of Health and Medical Research), UMR 1260, Regenerative Nanomedicine (RNM), FMTS, Strasbourg, France; 3 Intensive Care Department, Hospital Universitari Vall d’Hebron Universitat Autònoma de Barcelona, Barcelona, Spain

**Keywords:** Endotoxin, Extracorporeal circulation, Sepsis, Shock, Biomarker

## Abstract

Septic shock driven by endotoxemia is associated with high mortality despite advances in supportive care. Polymyxin B hemoperfusion (PMX-HP) selectively removes circulating endotoxin and has shown variable efficacy in randomized trials. While earlier studies, such as EUPHAS, suggested benefit, subsequent trials, including ABDO-MIX and EUPHRATES, yielded neutral results, partly due to heterogeneous patient selection. The recently completed TIGRIS trial addressed these limitations by enrolling septic shock patients with intermediate endotoxin activity (EAA: 0.60–0.89) and high multiple organ dysfunction syndrome (MODS > 9) using a Bayesian design. In 151 evaluable patients, PMX-HP achieved the primary endpoint, with a 95.3% posterior probability of 28-day survival benefit (adjusted odds ratio [OR]: 0.67; absolute risk reduction [ARR] 6.4%). At 90 days, mortality reduction was greater (ARR: 17.4%; adjusted OR: 0.54; > 99% posterior probability of benefit), corresponding to a number needed to treat of 8.1. These results support the targeted use of PMX-HP in a biomarker-defined subgroup and may facilitate broader regulatory approval.

## Introduction

Septic shock, a severe and often fatal condition, is characterized by overwhelming infection and systemic inflammation. Endotoxin, a component of Gram-negative bacterial cell walls, is a key mediator in the pathogenesis of sepsis and is associated with an increased risk of multiple organ failure and mortality [1]. Conventional antibiotic treatment targets and kills bacteria but does not necessarily neutralize endotoxin, which can be particularly problematic in systemic infections. This limitation has prompted the development of therapeutic strategies that not only eliminate bacteria but also aim to neutralize endotoxins, either within or outside the bacterial cells [2, 3]. While previous pharmacological attempts to neutralize endotoxin have failed [2], polymyxin B hemoperfusion (PMX-HP), a technique that removes endotoxin from the bloodstream using a specialized cartridge, has shown promise in preliminary studies. However, its efficacy has remained controversial, with mixed results depending on patient selection and study design. The development of PMX-HP emerged from the need to address the harmful effects of LPS in Gram-negative bacterial infections, particularly in the context of septic shock. While polymyxin B was originally developed as an antibiotic in the 1950s, its potent endotoxin-binding properties led to the exploration of its non-antibiotic applications [4]. In the late 1980s and early 1990s, Japanese researchers engineered a hemoperfusion cartridge in which polymyxin B was covalently immobilized on polystyrene fibers, enabling the selective adsorption of circulating endotoxins without systemic toxicity, and gained regulatory approval in Japan by 1993 [5]. Since then, PMX-HP has been used in over 360,000 units cases globally [6], especially in Japan and parts of Europe. Its mechanism involves direct removal of endotoxin from the bloodstream, thereby reducing systemic inflammation and stabilizing hemodynamics [7]. Over the past two decades, numerous studies, including the EUPHAS, ABDO-MIX, and EUPHRATES trials, have evaluated its efficacy, with mixed results. These findings have led to ongoing refinement of patient selection criteria and trial design, culminating in recent confirmatory studies such as the TIGRIS trial, aimed at securing broader international regulatory approval. This review introduces the history of PMX-HP research over 30 years and the current status of its application.

### Current status of clinical application

The hemoperfusion with PMX (Toraymyxin^™^, Toray, Tokyo, Japan) is currently available as clinical therapy in Japan and at least 14 European countries, including Italy, Spain, and others. Overall, Toraymyxin^™^ has been used in over 100,000 cases in Japan [6]. Toraymyxin^™^ is also available in Asian countries, including Korea and Taiwan, where it is approved for use in patients with endotoxic septic shock [8]. In contrast, routine regulatory approval is still pending in the United States**,** although the device holds Food and Drug Administration (FDA) distinction as an Investigational Device Exemption (IDE) for research and benefits from Breakthrough Device status [9]. In the United States, evidence from randomized controlled trials (e.g., ABDOMIX, EUPHRATES) has produced inconclusive or mixed results. Major limitations included inadequate patient selection and heterogeneity in trial design [10]. However, the therapy has gained renewed momentum following the FDA’s Breakthrough Device Designation and the completion of the TIGRIS trial, a Bayesian-designed Phase 3 study intended to clarify efficacy in endotoxin-positive patients [11]. Although PMX-HP has shown potential in reducing inflammation and improving hemodynamic stability, particularly when guided by biomarker-based selection such as an endotoxin activity assay (EAA) value of ≥ 0.6, its adoption outside of Asia remains limited due to the need for more robust clinical evidence and formal regulatory approval. This highlights the gap between promising mechanistic rationale and the stringent requirements of international regulatory agencies.

### EUPHAS trial

The effect of PMX-HP was first reported by the EUPHAS (Early Use of Polymyxin B Hemoperfusion in Abdominal Sepsis) randomized controlled trial (RCT) [12] (Table 1). This study evaluated the efficacy of adding PMX-HP to conventional therapy in patients with severe sepsis or septic shock following emergency surgery for intra-abdominal infections. Conducted in 10 Italian ICUs, the study enrolled 64 patients who were randomized to receive either standard therapy alone or standard therapy plus two sessions of PMX-HP. The primary outcomes were changes in mean arterial pressure (MAP) and vasopressor requirements at 72 h. Results showed that patients in the PMX-HP group experienced a significant increase in MAP and a reduction in vasopressor needs compared to the control group. Additionally, improvements in oxygenation (PaO_2_/FIO_2_ ratio) and organ dysfunction (Sequential Organ Failure Assessment [SOFA] score) were observed in the PMX-HP group. Notably, 28-day mortality was significantly lower in the PMX-HP group (32%) compared to the control group (53%), with an adjusted hazard ratio of 0.36. The study concluded that early use of PMX-HP in this targeted population improved hemodynamics, organ function, and survival, suggesting a potential benefit for selected patients with abdominal septic shock. However, this study was terminated early after a predetermined interim analysis. Although it was understandable, this decision has been criticized because the number of patients was too small, and early stopping can overestimate treatment effects and limit the robustness of the findings [13]. Therefore, a larger and blinded trial was demanded [14].

**Table 1:** Comparison of major polymyxin hemoperfusion trials

Trial	Year	Country/region	Design	Patient population	Key inclusion criteria	Primary endpoint	Sample size	Main results	Key remarks
EEUPHAS	2009	Italy	Multicenter RCT	Septic shock post-abdominal surgery	Confirmed Gram-negative infection; ongoing septic shock after surgery	28-day mortality	64	28-day mortality: 32% PMX versus 53% control (*p* = 0.049)	Stopped early due to benefit; small sample; all post-op abdominal sepsis
ABDO-MIX	2015	France	Multicenter RCT	Peritonitis from abdominal sepsis	Septic shock within 36 h of surgery; Gram-negative suspected/confirmed	28-day mortality	243	28-day mortality: 27.7% PMX versus 19.5% control (NS); no improvement in organ failure	No benefit; high cartridge clotting rate; more protocol deviations
EUPHRATES	2018	USA/Canada	Multicenter RCT	Septic shock with high endotoxin activity	EAA ≥ 0.60; on vasopressors; MODS ≥ 9	28-day mortality	450	Overall: no difference (PMX 37.7% vs. control 36.7%); subgroup with EAA 0.60–0.89 had ARR 10.7%	Largest trial; predefined high EAA subgroup showed signal of benefit
TIGRIS	2025	USA	Phase III multicenter RCT (Bayesian)	Endotoxic septic shock	EAA 0.60–0.89; MODS > 9; high organ dysfunction	28-day mortality	157 (2:1 randomization)	28-day: ARR 6.4% (OR 0.67; 95.3% posterior probability of benefit); 90-day: ARR 17.4% (> 99% posterior probability)	Confirmed benefit in biomarker-defined subgroup; incorporated prior EUPHRATES data; FDA PMA submission planned

RCT: randomized controlled trial, PMX: polymyxin B, EAA: endotoxin activity assay, ARR: absolute risk reduction, MODS: multiple organ dysfunction syndrome, OR: odds ratio, FDA: Food and Drug Administration, PMA: premarket approval

### ABDO-MIX study

The ABDO-MIX study was a multicenter, randomized controlled trial conducted in 18 French ICUs to evaluate whether early PMX-HP could reduce mortality and organ failure in patients with septic shock due to peritonitis following emergency surgery [15]. A total of 243 patients were randomized to receive either conventional therapy alone or conventional therapy plus two sessions of PMX-HP. The primary outcome, 28-day mortality, was 27.7% in the PMX-HP group versus 19.5% in the control group (*P* = 0.14), showing no statistically significant benefit. Similarly, 90-day mortality and reductions in organ failure (SOFA score) did not differ significantly between groups. Subgroup analyses failed to identify any group clearly benefiting from PMX-HP. The study concluded that early use of PMX-HP did not improve survival or organ function in this patient population and therefore cannot be recommended for septic shock secondary to peritonitis. The study has several limitations. First, the study was conducted in French ICUs, and results may not be directly generalizable to centers with different patient populations, standards of care, or resource availability. A high incidence of cartridge clotting and termination of the treatment is unusual and has not been seen in other studies. Indeed, only 81 of the 119 treated had completed two sessions of PMX-HP [16]. Second, the ABDO-MIX trial did not screen patients for elevated circulating endotoxin levels to identify those most likely to benefit from PMX-HP therapy. As a result, a substantial proportion of patients randomized to the PMX-HP group had low or negative endotoxin levels, potentially leading to a mismatch between treatment allocation and biological indication, thereby exposing some patients to the risks of therapy without a corresponding likelihood of benefit [17]. Other than that, although the study was a randomized trial, the lack of blinding could affect the results. The nature of the intervention made it impossible for investigators to remain blinded to treatment allocation, which may have introduced bias into the study results. Among all the limitations, poor adherence to the protocol seemed to be the biggest problem. Many of the patients in the intervention arm could not complete two sessions of hemoperfusion as planned, which should affect the per-protocol analysis [18]. Altogether, the trial illustrated both methodological and practical challenges of implementing PMX-HP in real-life critical care, particularly in Europe.

### EUPHRATES trial

The EUPHRATES trial was designed to rigorously evaluate whether PMX-HP could improve survival in patients with septic shock and high endotoxin activity [19]. This multicenter, randomized, double-blind, sham-controlled trial enrolled 450 critically ill adults with septic shock and elevated blood endotoxin levels (defined as an EAA ≥ 0.60). Patients were recruited from 55 tertiary hospitals in North America between September 2010 and June 2016, with follow-up through June 2017. Eligible participants had persistent arterial hypotension requiring vasopressors, received intravenous antibiotics, had substantial fluid resuscitation, and exhibited at least one new organ dysfunction. Exclusion criteria included limitations on treatment escalation or terminal illness precluding short-term survival. Participants were randomly assigned to receive either two sessions of polymyxin B hemoperfusion (each lasting 90–120 min) plus standard medical therapy, or sham hemoperfusion plus standard therapy. Treatments were administered within 24 h of enrollment. The sham procedure mimicked the actual treatment to maintain blinding of the clinical care team, with only the research staff aware of group assignments. The primary outcome was 28-day all-cause mortality, assessed both in the entire study population and in a subgroup with a MODS greater than 9, reflecting higher severity of illness. Secondary outcomes included survival time, changes in organ dysfunction, mean arterial pressure, urine output, and creatinine levels within 72 h. The results indicated that of the 450 patients enrolled (mean age 59.8 years; 39.3% women; mean acute physiology and chronic health evaluation [APACHE] II score 29.4), 449 completed the study. The 28-day mortality rate was 37.7% in the polymyxin B group and 34.5% in the sham group (risk difference 3.2%; 95% confidence interval [CI], − 5.7% to 12.0%; relative risk [RR], 1.09; *P* = 0.49), indicating no statistically significant difference. In the MODS > 9 subgroup, mortality rates were nearly identical (44.5% vs. 43.9%). Serious adverse events were common in both groups, with no significant differences in the types or frequencies of complications. The EUPHRATES trial found that adding PMX-HP to conventional therapy did not reduce 28-day mortality in patients with septic shock and high endotoxin activities. Despite rigorous design (double-blind, sham-controlled), the trial failed to show benefit, emphasizing the gap between pathophysiological plausibility and clinical outcomes in sepsis research [20].

Consequently, the post hoc analysis of the EUPHRATES trial showed potential benefits for patients with certain EAA levels [21]. Thus, the study was repeated in the following study (TIGRIS trial), and the data were added to the former trial (EUPHRATES). Using a precision medicine approach, eligibility criteria have been modified in the second study to include patients with a MODS score > 9 and EAA levels between 0.60 and 0.89 [22].

### Meta-analyses

Meta-analyses were performed to determine the effect of PMX-HP each time after the RCT. After the EUPHRATES trial, Fujii et al. [23] conducted a systematic review and meta-analysis to evaluate the efficacy and safety of PMX-HP in critically ill adults with sepsis or septic shock. Six randomized controlled trials (857 patients) were included. The primary outcomes assessed were 28-day all-cause mortality, serious adverse events, and organ dysfunction scores. The pooled risk ratio for 28-day mortality with PMX-HP was 1.03 (95% CI 0.78–1.36), indicating no significant mortality benefit. The risk of serious adverse events was higher in the PMX-HP group, though not statistically significant (RR 2.17; 95% CI 0.68–6.94). No significant improvement was observed in organ dysfunction scores. The certainty of the evidence was rated as low for both benefit and harm. Low certainty reflected limited sample sizes, lack of blinding in some trials, and strong heterogeneity across regions (notably Japan vs. Europe/North America). The review concluded that there is currently insufficient evidence to support the routine use of PMX-HP in sepsis or septic shock treatment.

Following this, Putzu et al. [24] evaluated 37 randomized controlled trials involving 2499 patients to assess whether extracorporeal blood purification techniques reduce mortality in sepsis and septic shock. PMX-HP, overall, was associated with a modest reduction in mortality (RR = 0.88; 95% CI, 0.78–0.98), although the certainty of this evidence was very low due to heterogeneity and small-study effects. They also examined the effects of hemofiltration and plasmapheresis. The results showed a borderline mortality benefit (RR = 0.79; 95% CI, 0.63–1.00) in hemofiltration, and a significant benefit (RR = 0.63; 95% CI, 0.42–0.96) in plasmapheresis, but both with very low certainty. Regional variations were notable, with studies from Asia showing more favorable outcomes. Overall, current evidence does not support the routine use of blood purification in sepsis and underscores the need for further high-quality trials.

Other than those, the latest meta-analysis by Chen et al. [25] evaluated 60 randomized controlled trials involving 4595 adult patients with severe infection or sepsis/septic shock to assess the efficacy of 16 extracorporeal blood purification modalities. The analysis found that plasma exchange (RR 0.61; 95% CI, 0.42–0.91) and PMX-HP (RR 0.70; 95% CI, 0.58–0.86) were associated with reduced mortality compared to standard care; however, the certainty of evidence was low for plasma exchange and very low for PMX-HP due to clinical heterogeneity and risk of bias. In this study, while the effect of PMX-HP was apparent, it was limited to high-mortality settings and older or region-specific studies, with its effect disappearing when higher-quality trials were isolated. Overall, current evidence does not support routine use of blood purification in sepsis and underscores the need for further high-quality trials. Future studies must be biomarker-driven, adequately powered, and harmonized internationally [26, 27].

### Other clinical studies

The EUPHAS 2 registry was a large, retrospective, multicenter study evaluating the real-world use of PMX-HP in 357 critically ill patients with severe sepsis or septic shock due to suspected or proven Gram-negative infections across 57 centers in Europe and Asia [28]. Most patients (85.4%) had septic shock, with abdominal (44%) and pulmonary (17.6%) infections being the most common sources. After PMX-HP, significant improvements were observed in cardiovascular, respiratory, and renal SOFA score components at 72 h. The overall 28-day survival rate was 54.5%, rising to 60.4% for abdominal and 64.5% for early-treated abdominal infections. Patients with marked cardiovascular improvement post-PMX-HP had a significantly higher 28-day survival (75% vs. 39%). European patients showed better survival than Asian patients, though populations differed in infection source and severity. The study confirmed PMX-HP’s feasibility and potential clinical benefits in sepsis, with no significant adverse events reported.

The *J*-SEPTIC study is a large, retrospective, multicenter study that originally examined the therapeutic effect of DIC and subsequently assessed whether PMX-HP improves survival in septic shock patients. Analyzing data from 1723 patients in Japanese ICUs, the study used propensity score matching to compare 262 patients treated with PMX-HP to 262 controls. Results showed significantly lower all-cause hospital mortality in the PMX-HP group (32.8% vs. 41.2%; OR 0.681, *P* = 0.042) and more ICU-free days in the first 28 days (median 18 vs. 14 days, *P* = 0.045). However, ICU mortality did not differ significantly. The findings suggest a potential survival benefit, but as with all retrospective studies, residual confounding and selection bias limit the strength of conclusions [29].

These observational studies support the feasibility, safety, and potential clinical benefit of PMX-HP in routine care for septic shock. While outcomes of the above studies appeared favorable, particularly with early and complete therapy, the retrospective nature limits interpretation. The registry reinforced the need for well-controlled, biomarker-guided randomized trials like the EUPHRATES and TIGRIS trials.

### Obstacles in PMX-HP study

Current clinical studies of PMX-HP face several key obstacles that have limited consistent demonstration of clinical benefit. One major issue is inadequate patient selection; many trials, including EUPHAS and ABDO-MIX, did not use circulating endotoxin levels to identify patients most likely to respond, resulting in the inclusion of individuals with low or negative endotoxin activity. This misalignment may have diluted treatment effects and exposed patients to unnecessary risks [30]. Additionally, heterogeneity in study design, such as variability in timing of treatment, illness severity, and supportive care, has made cross-study comparisons difficult [31]. Although some early studies suggested hemodynamic and organ function improvement, large randomized trials have failed to consistently demonstrate a survival benefit [32]. Meanwhile, the EUPHRATES trial had employed biomarker-guided strategies using tools, i.e., EAA, which could improve therapeutic targeting. On the other hand, statistical and regulatory complexities, particularly around newer methodologies like Bayesian trial design, add further challenges to the TIGRIS trial. Finally, beyond scientific issues, concerns over cost-effectiveness and logistical feasibility (rapid EAA testing, specialized equipment, trained staff) remain obstacles to broader implementation.

### TIGRIS trial

The TIGRIS trial is a Phase III, randomized, open-label study designed to evaluate the efficacy and safety of PMX-HP (Toraymyxin^™^-20R cartridge) in adult patients with endotoxemic septic shock. Its primary objective is to assess whether adding PMX-HP to standard care can reduce 28-day all-cause mortality. Secondary objectives include evaluating changes in mean arterial pressure and vasopressor requirements by Day 3, overall survival time up to 28 days, and 14- and 28-day mortality in subgroups with higher vasopressor needs. The trial builds on findings from the EUPHRATES trial by targeting a more refined patient population, specifically those with moderate endotoxin activity (EAA 0.6–0.89), and employs a Bayesian adaptive design to potentially validate the clinical benefit of PMX-HP and support its regulatory approval. In February 2025, Spectral Medical Inc. (Toronto, Canada) announced the completion of patient enrollment for its Phase 3 TIGRIS trial, a randomized controlled study evaluating PMX-HP in patients with endotoxic septic shock [33]. A total of 157 patients were enrolled, with 151 evaluable and 100 receiving PMX-HP treatment. PMX-HP is specifically designed to adsorb circulating endotoxins from the bloodstream. The TIGRIS trial, using a 2:1 Bayesian randomization design, serves as a confirmatory study to support FDA approval in the USA. Spectral has already submitted all non-clinical modules of its Premarket Approval (PMA) to the FDA and plans to submit the clinical data by October 2025 [34].

An early report from Spectral was released on Aug 12, 2025 [6]. In that report, the intention-to-treat analysis showed that the adjusted posterior probability of benefit for PMX at 28 days was 95.3%, exceeding the prespecified 95% target and meeting the primary endpoint. Adjusted OR was 0.67 (95% credible interval [CrI] 0.39–1.08). Observed 28-day mortality was 38.7% with PMX versus 45.1% with standard care, an absolute difference of 6.4%. Bayesian pooled unadjusted analysis showed an 8.3% absolute risk reduction (ARR) and an 18% relative risk reduction (RRR), with a 92.3% posterior probability of benefit. In the modified intention-to-treat population (patients receiving assigned treatment), mortality was 37.0% versus 45.1% (ARR = 8.1%).

The key secondary endpoint, 90-day mortality, showed > 99% posterior probability of benefit. Adjusted OR was 0.54 (95% CrI 0.32–0.87). Observed 90-day mortality was 43.4% for PMX versus 60.8% for controls, an ARR of 17.4%. The pooled Bayesian unadjusted ARR was 12.3% (RRR 20.2%), with a number needed to treat (NNT) of 8.1 to save one life at 90 days. Although promising, these findings remain preliminary and await full peer-reviewed publication. These results confirm earlier signals from the EUPHRATES and EUPHAS trials that PMX benefits a specific patient subgroup with intermediate endotoxin activity.

## Conclusion

The TIGRIS trial provides compelling evidence that PMX can offer a meaningful survival benefit when applied to a carefully selected subgroup of septic shock patients with intermediate endotoxin activity (EAA 0.60–0.89) and high organ dysfunction. While the 28-day mortality reduction (absolute difference 6.4%, adjusted OR 0.67) narrowly missed conventional statistical significance, the Bayesian analysis incorporating prior EUPHRATES data demonstrated a > 95% posterior probability of benefit. Notably, the effect size was greater at 90 days, with an absolute mortality reduction of 17.4% and > 99% posterior probability of benefit, suggesting that PMX may exert its most important impact on longer-term survival, which is clinically relevant in modern critical care where patients often survive beyond the acute phase.

These findings reinforce the concept that PMX is not a universal therapy for all sepsis cases, but may provide benefit in a biomarker-defined high-risk subgroup. However, the relatively small sample size (*n* = 157) and the need for rapid EAA testing and strict patient selection highlight practical barriers to broad implementation, and real-world studies will be essential before PMX-HP can be integrated as the standard of care.

## Data Availability

No datasets were generated or analyzed during the current study.
